# Brain mechanisms associated with facial encoding of affective states

**DOI:** 10.3758/s13415-023-01114-3

**Published:** 2023-06-22

**Authors:** Miriam Kunz, Jen-I Chen, Stefan Lautenbacher, Pierre Rainville

**Affiliations:** 1 Department of Medical Psychology and Sociology, University of Augsburg, Augsburg, Germany; 2 Bamberger Living Lab Dementia (BamLiD), University of Bamberg, Bamberg, Germany; 3 Centre de recherche de l’Institut universitaire de gériatrie de Montréal (CRIUGM), Université de Montréal, Montréal, Canada; 4 Department de stomatologie, Faculté de médecine dentaire, Université de Montréal, Montréal, Canada

**Keywords:** Facial expression, Facial display, Facial affect, Affect communication, Nonverbal communication, Brain, Neural mechanisms

## Abstract

Affective states are typically accompanied by facial expressions, but these behavioral manifestations are highly variable. Even highly arousing and negative valent experiences, such as pain, show great instability in facial affect encoding. The present study investigated which neural mechanisms are associated with variations in facial affect encoding by focusing on facial encoding of sustained pain experiences. Facial expressions, pain ratings, and brain activity (BOLD-fMRI) during tonic heat pain were recorded in 27 healthy participants. We analyzed facial expressions by using the Facial Action Coding System (FACS) and examined brain activations during epochs of painful stimulation that were accompanied by facial expressions of pain. Epochs of facial expressions of pain were coupled with activity increase in motor areas (M1, premotor and SMA) as well as in areas involved in nociceptive processing, including primary and secondary somatosensory cortex, posterior and anterior insula, and the anterior part of the mid-cingulate cortex. In contrast, prefrontal structures (ventrolateral and medial prefrontal) were less activated during incidences of facial expressions, consistent with a role in down-regulating facial displays. These results indicate that incidences of facial encoding of pain reflect activity within nociceptive pathways interacting or possibly competing with prefrontal inhibitory systems that gate the level of expressiveness.

## Introduction

With the growing impact of the affective sciences (Dukes et al., 2021), the interest in facial expressions is regaining widespread attention. Facial expressions are thought to play an important role in social interactions by rapidly informing observers about the inner affective state of the expressor (Fridlund et al., 1997). In the case of negative affective states, such as disgust, sadness, or pain, facial expressions can elicit empathy in the observer and thereby facilitate social support (Craig et al., 2011; Decety et al., 2008; Kunz et al., 2018). The encoding of affective states via facial muscle movements has been attributed to innate predispositions (Darwin, 1872/2005; Izard, 1994; Ekman, 1999). Nevertheless, the incidence with which affective states are accompanied by facial expressions is rather unstable, which has nourished animated debates on how well facial expressions really mirror the underlying affective state (Parkinson 2005; Reisenzein et al., 2013; Ruch, 1995; Durán and Fernández-Dols, 2021; Barret et al., 2019). Manyfold reasons for these variations in facial affect encoding have been suggested, spanning from personal characteristics (e.g., gender, cognitive status (Kunz et al., 2006, 2007; McDuff et al., 2017, Seidl et al., 2012)) to contextual and cultural factors (e.g., social context, social display rules (Jakobs et al., 2001; Karmann et al., 2014; Kappesser, 2019; Hess et al., 1995). Even within the same person and keeping contextual factors stable, there are still great variations in the degree to which affective states are accompanied by overt facial expressions (Craig et al., 2011; Reisenzein et al., 2013). So far, it is difficult to predict when an affective state is facially expressed and what mechanisms underlie these incidences of facial affect encoding.

In a previous study, we investigated whether neural mechanisms might help to explain variance in facial encoding. In this study, we used phasic experimental pain and analysed which neural mechanisms might underlie the facial encoding of pain (Kunz et al., 2011). Experimental pain is an ideal model to study variations in facial affect encoding for several reasons. First, experimental pain activates well-defined nociceptive pathways and brain networks (Treede et al., 1999; Apkarian et al., 2005; Dum et al., 2009; Duerden and Albanese 2013; Wager et al., 2013). Second, it is possible to elicit comparable levels of subjective (pain) experiences across time, to repeat stimuli without substantial habituation, and to elicit strong sensations that make the occurrence of facial expressions more likely (Craig et al., 2011). Using phasic experimental pain and comparing trials with and without facial expressions of pain, we found that the occurrence of facial expressions was associated with increased activity in pain-related areas as well as with an activity decrease in prefrontal areas (Kunz et al., 2011). The activity decrease in prefrontal areas is in line with the notion that the prefrontal cortex plays a crucial role in regulating facial affect encoding by acting as an output gating system (Karmann et al., 2016).

Comparing very brief painful events with and without facial expressions was a first step into studying variations in facial affect encoding and its underlying neural mechanism. However, pain and other affective states are typically more sustained experiences, with variations in facial affect encoding referring to epochs with and without facial displays occurring across the sustained experience. Thus, to increase ecological validity, experimental models of more sustained or tonic experiences of pain should be used to investigate how brain activity changes when facial affect encoding occurs. Tonic pain models also have the advantage that possible confounds by novelty due to abrupt onset and offsets of noxious stimulation can be eliminated.

To this purpose, we experimentally induced tonic heat pain (2-minute duration) and recorded facial expressions and brain activation (functional MRI) to investigate neural mechanisms associated with incidences of facial displays of pain.

## Materials and methods

### Participants

Twenty-seven, healthy volunteers (female: N = 14, male: N = 13) aged 18 to 30 years (mean age 22.7 years; SD = 3.2) participated in the study and were recruited via advertisements posted on the campuses of the Université de Montréal and McGill University. All participants were included in a previously published report on a phasic pain paradigm (Kunz et al., 2011), but the present study is based on a separate dataset involving tonic heat pain. None had taken any analgesic medication or alcohol for at least 24 hours before the test session. Exclusion criteria included all acute or chronic diseases. Furthermore, individuals who proved facially stoic in a 30-min, pre-experimental session with the same experimental protocol also were excluded, which resulted in a reduced sample size compared with the sample reported in Kunz et al., 2011). Of the 27 participants who took part in the present study, four participants had to be excluded from the analyses due to technical difficulties with the video recording (n = 1), lack of facial expression (n = 1), or excessive head movement (n = 2), resulting in 23 participants (female: n = 13, male: n = 10). The consent form indicated that the purpose of the study was to investigate the cerebral response to painful stimulation and mentioned that they would be filmed during the experiment, although this was not emphasized at any point during the study. The study protocol was approved by the ethics committee of the Centre de recherche de l’Institut universitaire de gériatrie de Montréal and all participants gave written, informed consent.

### Materials and procedure

**Fig. 1:**
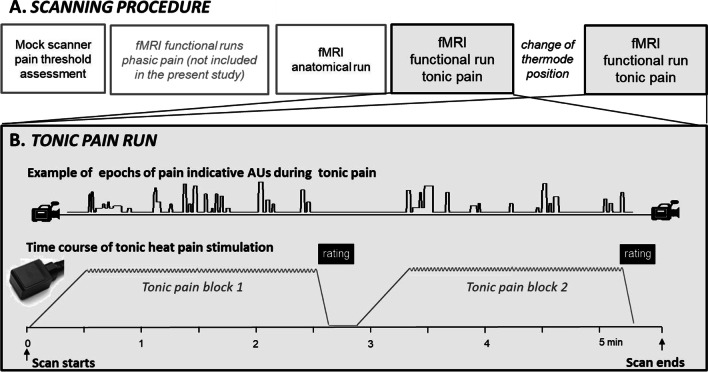
Study procedures: **A**. Overview of the whole experiment with its different imaging runs; **B**. Depiction of the tonic pain runs

An overview of the experimental design is given in Fig. 1. The present article focuses on the two functional runs of tonic pain stimulation.

#### Stimulation

Pain was induced experimentally by an MRI-compatible, Peltier-based, thermal stimulator with a 3 × 3 cm^2^ contact probe (Medoc TSA-2001; Medoc Ltd, Ramat Yishai, Israel). The contact probe was attached to the left lateral lower leg. Baseline temperature was always set to 38 °C. To ensure that temperature intensities were perceived as painful but tolerable in all participants (to prevent floor as well as ceiling effects), temperature intensities were tailored to the individual pain threshold. Following a familiarization trial, heat pain thresholds were determined by using the method of adjustment in a mock MRI scanner immediately before the scanning session (the average of 5 trials was used as the threshold estimate). Participants were brought into the scanner room for the experiment immediately after.

*Tonic heat stimuli* were administered according to protocol of the Tonic Heat Pain Model (Fig. 1) (Lautenbacher et al., 1995). A 2-minute stimulus was applied twice in each of the two functional scans (Fig. 1), resulting in 2 functional runs x 2 stimuli x 2-minute stimulation phases = 8 minutes of tonic pain stimulation. The temperature increased from baseline (38 °C) with a heating rate of 0.5 °C/s to reach the preset target temperature corresponding to the individual pain threshold, as determined before the experiment. After target temperature was reached, the 2-minute stimulation phase started with repeated heat pulses of +1.3 °C above pain threshold administered during the 2-minute plateau at a constant rate of 30 pulses per minute. These stimulation parameters were previously shown to produce a continuous and stable experience of pain (Lautenbacher et al., 1995). To avoid local sensitization, the site of heat stimulation was changed between the two functional runs.

#### Self-report ratings

After each tonic stimulus, participants were asked to rate the overall intensity and unpleasantness of the 2-minute stimulation phase on a computerized VAS-scale displayed using E-Prime (Psychology Software Tools Inc.) and converted linearly to values between 0 and 100. The VAS for sensory intensity of pain was labeled with verbal anchors from “no pain” (0) to “extremely strong pain” (100). Pain unpleasantness was labeled with “no pain” (0) to “extremely unpleasant pain” (100). All participants were instructed about the conceptual distinction between sensory intensity of pain and pain unpleasantness following the instructions of Price et al. (1983). VAS sensory and unpleasantness scales appeared successively and were displayed using E-Prime (Psychology Software Tools Inc.) and projected on a screen located at the head-end of the scanner and viewed by the subjects via a mirror attached above the head coil. The ratings were done by moving a computer-controlled cursor using the index and middle finger of the right hand and were recorded in E-Prime.

#### Facial expressions of pain

During both functional scans, the face of the subject was videotaped using a small MRI-compatible camera (MRC Systems, Heidelberg, Germany) mounted onto the head coil. The camera was carefully positioned to capture the face of the subject reflected through a mirror attached above the head coil, without blocking the visual field of the subject. The onset of each thermal stimulus was marked automatically on the video recording by using a signal sent from the stimulator to the sound card. A software designed for the analysis of observational data (Observer Video-Pro; Noldus Information Technology) was used to segment the videos and to score facial expressions into a time-related database. Time segments of 2 minutes beginning with stimulus reaching the target temperature were selected for scoring of facial expressions. We quantified facial expressions using the Facial Action Coding System (FACS; Ekman and Friesen, 1987), a fine-grained, anatomically based system considered to be the “gold standard” when decoding facial expressions, including the facial expression of pain (Craig et al., 2011). The FACS is based on the anatomical analysis of facial movements and distinguishes 44 different Action Units (AUs) that are produced by a single muscle or a combination of muscles. Two certified FACS coder identified the onset and offset of all AUs. Five percent of the video segments were coded by both observers and interrater reliability was 0.87, as calculated using the Ekman–Friesen formula (Ekman and Friesen, 1987). The occurrence of AUs was sampled in bins of 200 msec for analysis.

**Fig. 2:**
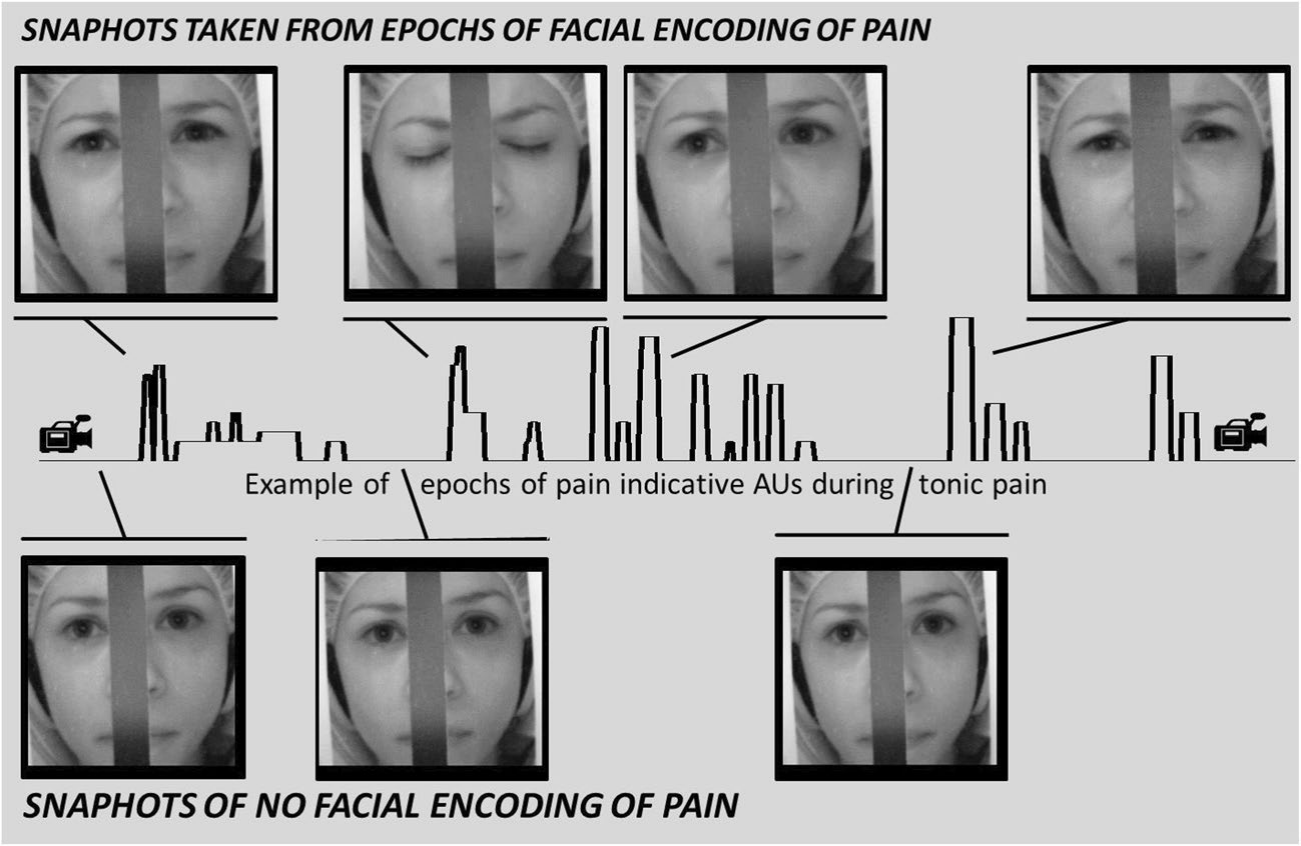
Facial expressions occurring during tonic pain stimulation. The upper half shows snapshots taken from epochs of facial encoding of pain; the lower half shows snapshots where no facial expressions of pain occurred

Moreover, AUs indicative of pain (Kunz et al., 2019), namely AU4 (corrugator muscle), AU 6_7 (orbicularis oculi muscle), AU 9_10 (levator muscle), and AU25_26_27 (orbicularis oris muscle) were combined (averaging) to form one pain-indicative facial expression variable that was then transformed into a dichotomous variable reflecting the presence (pain indicative AUs > 0) or absence of pain expression. Thus, we could divide the 8 minutes of tonic painful stimulation for each participant into epochs where facial expressions of pain occurred and epochs where the experience of pain was not accompanied by facial expressions of pain (examples are given in Fig. 2). It is important to note that this approach allows us to investigate epochs composed of any given combinations of pain indicative AUs or even being composed of a single pain indicative AU.

#### fMRI image acquisition and analyses

Imaging data were acquired at the “Unité de Neuroimagerie Fonctionnelle” of the “Centre de recherche de l’Institut de gériatrie de Montréal” using a 3T Siemens Magneton TIM Trio magnetic resonance imaging (MRI) system with a 12-channel head coil. Participants were placed in a comfortable position and their head stabilized with foam pads and headphones. Earplugs were also given to reduce the noise from the scanner. The two functional runs were preceded by one high-resolution, anatomical scan of 9 minutes. A total of 110 whole-brain volumes were acquired during each functional scan using blood oxygen level-dependent (BOLD) contrast. Each functional volume comprised 40 interleaved axial slices of 3.40-mm thickness parallel to the AC-PC line (in-plane resolution 3.44 x 3.44 mm). Volumes were acquired by using a gradient echo, echo-planar (EPI) T2*-weighted sequence (TR = 3,000 ms, TE = 30 ms; flip angle = 90°; matrix = 64 x 64; FOV = 220 x 220 mm^2^; bandwidth = 2,440 Hz/Px). Structural images were acquired by using a high-resolution, T1-weighted MP-RAGE sequence (TR = 2,300 ms, TE = 2.91 ms; flip angle = 9°; FOV = 256 mm; matrix = 256 × 240; 1- × 1- × 1.2-mm voxels; bandwidth = 240 Hz/Px; 160 slices per whole-brain volume).

Image analysis was performed by using SPM12 (Statistical Parametric Mapping, Version 12; Wellcome Department of Imaging Neuroscience, London, UK), executed in Matlab 8.6 (Mathworks, Sherborn, MA). Functional images were first pre-processed with slice-time correction, and motion corrected by realigning all images to the first image using six-parameter rigid body transformation and reslicing with fourth-degree B-spline interpolation. Additionally, images were preprocessed with SPM’s extension toolbox ArtRepair (v.5b3) to detect and repair by interpolation, outlier volumes before coregistraton and spatial normalization (Mazaika et al., 2007, 2009). The six motion parameters were condensed into a single summary statistic to calculate the root mean squared head position change (RMS movement) to assess subject head motion (Power et al., 2012). All remaining participants stayed well within half a voxel size (mean = 0.57 mm; SD = ±0.34). Instantaneous frame-wise displacement (FD) (Power et al., 2012) also was computed in ArtRepair by taking the sums the absolute values of volume-by-volume changes in the six rigid body parameters. The time-series of motion correction parameters were carefully examined for each individual run and led to the complete exclusion of two participants who showed excessive instantaneous movements (> voxel size). The average FD across remaining runs was 0.13 mm (SD = ±0.08), well below the recommended 0.5-mm threshold (Power et al., 2012). The BOLD images were then coregistered with the structural image and spatially normalized to MNI space by using the unified segmentation-based method, with the normalization parameters determined during the segmentation of the structural images. Spatial smoothing with 6-mm, isotropic, full-width, half maximum (FWHM), Gaussian kernel was subsequently applied to the functional images to increase signal-to-noise ratio. Given that there is a low frequency component to the probability of occurrence of facial responses during the 120-sec tonic stimulation versus the interstimulus baseline (Fig. 1B), a high-pass, temporal filter with a cutoff = 256 s was chosen to reduce the risk of removing meaningful variance in brain activity related to the facial response. Moreover, correction for autocorrelation between successive volumes were applied to the time series (AR1).

### Statistical analysis

Functional MRI images were analyzed by using the general linear model (GLM) in SPM12 to identify brain regions showing significant changes in BOLD signal (Ogawa et al., 1992). Subject-level statistical maps were modeled by using a canonical hemodynamic response function to obtain voxel-by-voxel parameter estimates of stimulus-related activity for each condition and each subject. We conducted an “epoch-by-epoch” analysis where the occurrence of each epoch of facial encoding of pain during the tonic painful stimulation were modelled as a regressor in the design matrix. The entire dataset contained 586 epochs.

To further account for the possible effect of head movements during the scans, the 6 motion correction parameters (3 translational and 3 rotational) were included in the design matrix as nuisance regressors. Additionally, mean signals across voxels from the white matter and the cerebrospinal fluid were added as covariates of no interest to remove possible physiological noise.

Contrast images showing areas of the brain associated with epochs of facial expressions of pain during tonic pain were generated for each subject and were subsequently used in second-level analyses to compute group average map by using one-sample *t*-test. Significance was determined using the False Discovery Rate method applied at the voxel level (FDR q = 0.05).

## Results

The mean (±SD) pain threshold was 45.58 °C (± 0.85). The tonic stimulation (target intensity +1.3 °C above threshold) was rated with an average VAS-intensity score of 78.3 (± 10.7) and an average VAS-unpleasantness score of 83.9 (± 9.9).

### Facial encoding of pain

During the 480 seconds of painful stimulation, facial expressions of pain were displayed on average across 142.1 seconds. Thus, approximately 30% of the sustained pain experience was accompanied by facial encoding of pain. Breaking the occurrence of facial encoding of pain down into discrete epochs, we found that participants on average displayed 26 epochs of facial expressions of pain across the 480 seconds of painful stimulation; each epoch lasted on average for 5.36 seconds (SD = 9.12; MIN = 0.20; MAX = 104.60 seconds). Examples of facial encoding of pain occurring during the tonic pain stimulation as well as the lack thereof are shown in Fig. 2.

### Neural correlates of facial encoding of pain

**Fig. 3:**
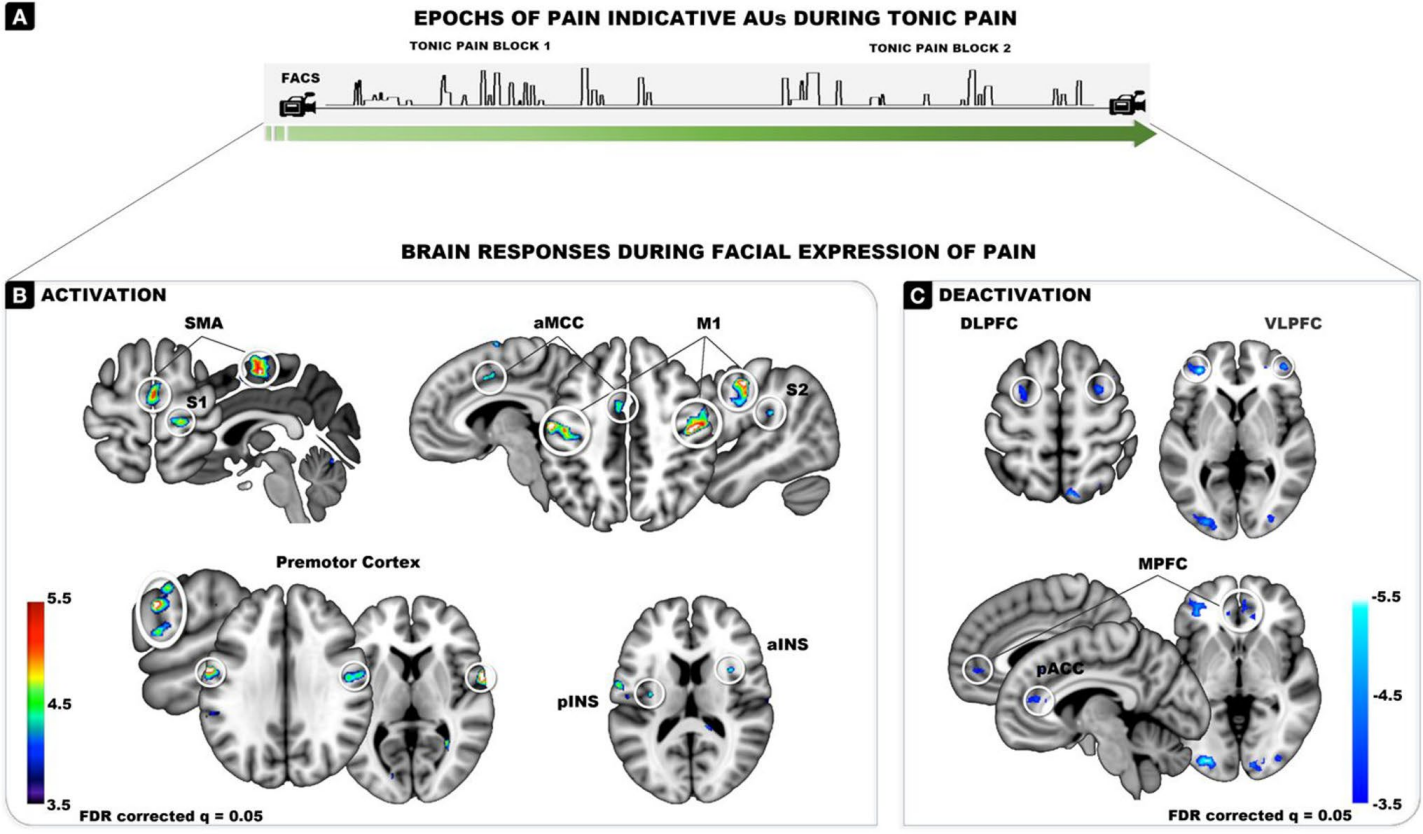
Brain activation (**B**) and deactivation (**C**) being associated with epochs of facial expressions of pain. (**A**) Example of these epochs occurring across the tonic pain stimulation

Positive brain activations significantly associated with epochs of facial encoding of pain during tonic pain stimulation (Fig. 3A) (FDR q = 0.05) were found in supplementary motor area (SMA), bilateral premotor (PMd), and the putative face area of the primary motor cortex (M1, bilateral) (Table 1; Fig. 3B), consistent with facial motor activity. Significant activation also was observed in pain-related cortical areas whenever pain was encoded in facial expressions during tonic heat pain. Peaks were found in the putative leg area of the primary somatosensory cortex (S1), anterior part of the mid-cingulate cortex (aMCC), the parietal operculum, and both the anterior and posterior insula (aINS, pINS).

**Table 1:** Brain activation and deactivation during epoch of facial encoding of pain

BRAIN AREA	COORDINATES			LOCAL PEAK t-value
	***x***	***y***	***z***	
***Activation***				
S1	-16	-28	70	3.82
	20	-30	62	5.05
M1	-56	-12	42	6.33
	56	-10	46	5.99
	-40	-18	38	6.08
	48	-12	46	6.07
Premotor	-60	-2	30	6.15
	60	-10	26	5.03
BA 6 Premotor	62	4	6	6.14
SMA	0	-10	60	5.71
aMCC	-6	0	46	4.55
S2	48	-34	24	4.43
aINS	34	8	14	4.41
pINS	-34	-12	14	4.51
	36	-14	12	4.00
Thalamus	-18	-14	0	3.80
***Deactivation***				
DLPFC	-28	2	58	4.35
	30	4	56	4.52
VLPFC	40	36	14	4.56
	-32	46	2	5.56
	42	48	4	5.04
MPFC	6	44	-2	4.16
Superior parietal	12	-74	56	5.14
Pregenual ACC	-6	38	14	4.22
	-6	34	38	4.22
Inferior temporal	-50	-62	-8	4.66
V1	-28	-88	-2	5.65
	22	-88	-10	5.34

FDR q = 0.05

In contrast, peaks of significant decrease in BOLD (FDR q = 0.05) were observed in the frontal areas ranging from dorsolateral to ventrolateral and medial prefrontal areas (Table 1; Fig. 3C).

## Discussion

The purpose of the present study was to investigate which neural mechanisms may underly variations in facial affect encoding by focusing on facial expressions during tonic pain stimulation. The data presented were obtained using a subsample of a study where we assessed facial responses to phasic heat pain (Kunz et al., 2011). Inducing tonic heat pain, we found that 1) the sustained experience of moderate to strong pain intensities was only partially accompanied by epochs of facial expressions of pain and 2) the occurrence of these epochs was associated with higher activation in motor- and pain-related areas of the brain, and with reduced activity in prefrontal areas.

### Facial encoding of pain

Despite the application of tonic heat stimulation of moderate to strong pain intensities, facial expressions were only partially displayed during these experiences. Indeed, the tonic pain stimulation was encoded in visible facial expressions of pain in only 30% of the time on average. Moreover, the facial encoding of pain across the 4 x 2 minutes of tonic pain stimulation occurred on average in epochs of 5 seconds, albeit with great variability within and between individuals. These variations in facial encoding of pain are well in line with the bulk of previous evidence that also showed rather low degrees of co-occurrence between affective states and facial expressions (Parkinson 2005; Reisenzein et al., 2013; Ruch, 1995; Durán and Fernández-Dols, 2021; Barret et al., 2019). It has been previously assumed that this dissociation between the affective state and its facial encoding might be due to insufficient emotion intensity (Reisenzein et al., 2006). However, given that our stimuli were rated as moderately/strongly painful (VAS ratings of 78 and 84 (out of 100) for intensity and unpleasantness, respectively) and given that we kept the stimulation stable across the 8 minutes, we can exclude that an insufficient stimulus intensity is the reason that the experience of pain often did not co-occur with facial expressions of pain. Congruently, the hypothesis that insufficient intensity is the reason for the low co-occurrence between affective state and its facial display also has been refuted for other affective states (e.g., surprise, sadness) (Reisenzein et al., 2013). Nevertheless, spontaneous fluctuations in central nociceptive processes and motor gating may explain such apparent mismatch.

### Neural activation associated with incidences of facial encoding of pain

During epochs of facial encoding of pain, we found increased activation in the primary motor cortex (M1; face area, bilateral) and premotor areas, conforming with previous findings (Bair 2003; Kunz et al., 2011, 2020, Morecraft e al., 2004; Vachon-Presseau et al., 2016). Besides these neural motor correlates, epochs of facial encoding of pain also were accompanied by higher activation in cortical targets of the spinothalamic pathways implicated in the processing of pain (Duerden and Albanese 2013, Wager et al., 2013). These included the primary somatosensory area (S1, leg area), the parietal operculum, the posterior and anterior insula, as well as the anterior part of the mid-cingulate cortex. This observation is in line with our previous findings that showed that the occurrence of facial expressions of pain to phasic pain stimuli is associated with higher activity in pain-related areas (Kunz et al., 2011).

The association between S1 activity and the occurrence of facial expressions of pain may reflect spontaneous fluctuations in the intensity of the pain experienced (independently of any change in the noxious input). This interpretation derives from several previous studies demonstrating that changes within S1 are closely associated with changes in the subjective pain intensity (Bushnell et al., 1999; Hofbauer et al., 2001; Kunz et al., 2020). The responses observed in the parietal operculum and posterior insula also are particularly relevant, because these areas constitute the main sites where intracerebral electrical stimulation can elicit pain, suggesting a critical causal role in pain perception (Mazzola et al., 2012; Ostrowsky et al., 2002). The enhanced nociceptive activity in anterior insula and the mid-cingulate cortex during epochs of facial expressions also is consistent with a role for these regions in saliency (Legrain et al., 2011) and threat-related facilitation of pain (Wiech et al., 2010). Incidences of facial encoding of pain may thus capture transient periods of enhanced nociceptive, saliency, and threat processing within pain-relevant cortical areas.

The aMCC represents a particularly interesting area in the context of tonic stimulation where participants are instructed to tolerate moderate-to-strong pain for several minutes. In addition to the nociceptive input received from the spinal cord through the mediodorsal nucleus, the aMCC is involved in regulating motor and behavioral responses (Dum et al., 2009). This region is considered a key interface between pain and adaptive regulatory processes involved in affect, cognition, and facial expression (Shackman et al., 2011). In humans, electrostimulation of the aMCC has been associated with eliciting simple movements or atonia of the hand, arm, or leg but also more complex goal-oriented actions (Carucana et al., 2018). Parvizi et al. (2013) provides detailed descriptions of experiential reports of two patients undergoing electrostimulation of the aMCC and demonstrates a role in motivational drives. For example, one patient reported “it was more of a positive thing like…push harder, push harder, push harder to try and get through this…” (p. 1360). Interestingly, the second patient also described the experience of a challenge to be overcome but added a social dimension to the experience: “Yeah…I don’t feel like there’s nothing I can do about it. I feel like…’cause I have to fight it, you know? I have to make it through […] I feel like if I give up, then I’ve let everybody else down” (see Table 1 in Parvizi et al., 2013). Willfully enduring moderate-to-strong tonic pain in an experimental context may generate similar experiences. Thus, in addition to the encoding of nociceptive brain processes, epochs of facial expressions of pain may communicate dynamic fluctuations in such motivational drive and socioaffective processes.

### Neural deactivation associated with incidences of facial encoding of pain

Facial expressions of affective states are under the control of complex regulatory processes. The present results replicate our previous findings showing that facial encoding of pain is accompanied by a decrease in prefrontal activation (Kunz et al., 2011, 2020). Repetitive transcranial magnetic stimulation (rTMS) (Karmann et al., 2016) further corroborated that facial encoding of pain is indeed intensified when the excitability of the prefrontal areas is experimentally decreased. These findings suggest that the prefrontal areas act as an output gating system that regulates the degree to which affective states are facially encoded.

The regulation of facial expressions is believed to be socially learned. Facial expressions of pain are rather stimulus-driven and reflexive during the first months of life, and children gradually learn to regulate their facial expressions according to social display rules (Larachotte et al., 2006). This is accompanied by a continual maturation of the prefrontal cortex and the white matter from early childhood to late adolescence (Yakovlev and Lecours, 1967; Paus et al., 1999; Lebel et al., 2019). Thus, the maturation of prefrontal areas and their efferent projections might facilitate or promote the development of gating control over facial expression. Our findings corroborate the notion that facial encoding of pain is generally suppressed by prefrontal systems and epochs of facial expressions of pain occur when the frontal gating is released.

In summary, the neural activation and deactivation found in association with facial encoding of pain imply that spontaneous fluctuations in nociceptive activity and endogenous motivational factors may interact or even compete with prefrontal inhibitory systems to regulate facial expressions.

### Limitations

In the present study, facial expression and neural activity were assessed continuously across tonic stimulation, whereas self-report was only assessed at the end of each 2-minute stimulus. Thus, we cannot determine to which degree facial encoding of pain was linked to changes in pain intensity ratings across tonic stimulation. However, given the low associations found between facial and subjective responses (Kunz et al., 2011), we are confident that our findings cannot simply be explained by changes in perceived pain intensity. Moreover, we used experimental heat stimuli to induce tonic pain sensations. Although the experimental model has many advantages (e.g., controllable level of noxious input), a direct generalization to clinical pain conditions cannot be assumed. Furthermore, the present data are taken from the same group of participants included in our previous study on facial responses to phasic pain (Kunz et al., 2011) and thus does not provide completely independent evidence for the neural encoding of facial expressions of pain. Nevertheless, in view of the current gap in the brain imaging literature on the brain processes involved in the encoding of affective states in facial expression, we submit that the present results provide valuable information on this important question.

## Conclusions

Even the experience of moderate-to-strong pain intensities does not lead to a constant visible facial display of pain. In incidences where pain was facially displayed, we found heightened thalamo-cortical nociceptive processing in combination with a deactivation of prefrontal structures. This implies that incidences of facial displays of pain reflect nociceptive activity and possibly endogenous motivational factors interacting or even competing with prefrontal inhibitory systems.

## Data Availability

The group activation maps reported in the study (excluding the video recordings showing the face of the participants) are available upon direct request to the corresponding author.
